# Comparison of IBD-related outcomes in patients with obesity treated with GLP-1 receptor agonists versus bariatric surgery

**DOI:** 10.1093/crocol/otag027

**Published:** 2026-04-16

**Authors:** M Housam Nanah, Arjun Chatterjee, Hisham Wehbe, Miguel Regueiro

**Affiliations:** Department of Internal Medicine, Cleveland Clinic Foundation, Cleveland, OH, United States; Department of Gastroenterology, Hepatology, and Nutrition, Digestive Disease Institute, Cleveland Clinic, Cleveland, Ohio, United States; Department of Gastroenterology, Hepatology, and Nutrition, Digestive Disease Institute, Cleveland Clinic, Cleveland, Ohio, United States; Department of Gastroenterology, Hepatology, and Nutrition, Digestive Disease Institute, Cleveland Clinic, Cleveland, Ohio, United States

**Keywords:** IBD, bariatric surgery, GLP1 RA, weight loss

## Abstract

**Introduction:**

The global prevalence of obesity is rising, paralleled by an increase in IBD (inflammatory bowel disease). While studies examining the impact of obesity on IBD have yielded conflicting results, some suggest that obesity may increase adverse outcomes, whereas BS (bariatric surgery) has been associated with improved IBD outcomes in patients with obesity. Glucagon-like peptide-1 receptor agonists (GLP-1 RAs) have emerged as a less invasive alternative to BS for managing obesity, with recent studies indicating potential benefits for IBD outcomes. However, GLP-1 RAs have not been directly compared with BS in patients with obesity and IBD.

**Methods:**

We conducted a retrospective cohort analysis using TriNetX, a database aggregating data from over 200 healthcare organizations across the United States. Adult patients with IBD were identified using ICD-10 codes. The cohort was divided into two groups: patients with obesity and IBD prescribed GLP-1 RAs (dulaglutide, semaglutide, liraglutide, lixisenatide, exenatide, albiglutide, or tirzepatide) who had not undergone BS, and patients with obesity and IBD who underwent BS but had not received GLP-1 RAs. To ensure active exposure, GLP-1 RA use required at least two prescriptions separated by at least 1 month. Propensity score matching was performed based on demographics, comorbidities, and IBD medications. Categorical variables were compared using chi-square tests, and continuous variables were compared using independent *t*-tests. Effect estimates were reported as odds ratios (ORs) with 95% confidence intervals (CIs), with statistical significance defined as *P* < .05.

**Results:**

A total of 17142 patients with obesity and IBD were identified, including 12965 patients treated with GLP-1 RAs and 4177 patients who underwent BS. Patients in the GLP-1 RA group were older at index (56.7 vs 52.9 years). They also had higher rates of comorbidities, including diabetes (64.3% vs 31.5%), chronic kidney disease (19.6% vs 14.1%), and nicotine dependence (20.4% vs 19.1%), but lower rates of alcohol use disorder (2.3% vs 4.6%), compared with the BS group. The GLP-1 RA group also had higher baseline use of IBD-related medications, including corticosteroids, immunomodulators, and biologic therapies. After propensity score matching, 3478 patients were included in each cohort. Despite a higher post-intervention BMI (body mass index) (34.5 vs 33.4 kg/m^2^; *P* < .0001) and shorter follow-up, patients treated with GLP-1 RAs demonstrated significantly lower odds of lower extremity deep venous thrombosis (OR 0.24, 95% CI, 0.16-0.35), pulmonary embolism (OR 0.27, 95% CI, 0.17-0.41), Clostridioides difficile infection (OR 0.22, 95% CI, 0.14-0.34), intestinal obstruction (OR 0.21, 95% CI, 0.15-0.29), abdominal pain (OR 0.34, 95% CI, 0.28-0.42), nausea (OR 0.42, 95% CI, 0.35-0.50), constipation (OR 0.44, 95% CI, 0.37-0.53), colectomy (OR 0.13, 95% CI, 0.09-0.20), and IBD flares (OR 0.44, 95% CI, 0.37-0.54), all *P* < .0001, compared with BS. Initiation of advanced IBD therapies was also lower in the GLP-1 RA group (OR 0.45, 95% CI, 0.33-0.61; *P* < .0001).

**Conclusion:**

Despite a higher burden of comorbidities and a greater mean BMI, patients with obesity and IBD treated with GLP-1 RAs experienced significantly fewer gastrointestinal complications, thromboembolic events, disease flares, and surgical interventions compared with those undergoing BS. These findings suggest that GLP-1 RAs may represent a viable, less invasive therapeutic option for weight management and disease modulation in patients with obesity and IBD.

## Graphical Abstract

**Graphical Abstract:**
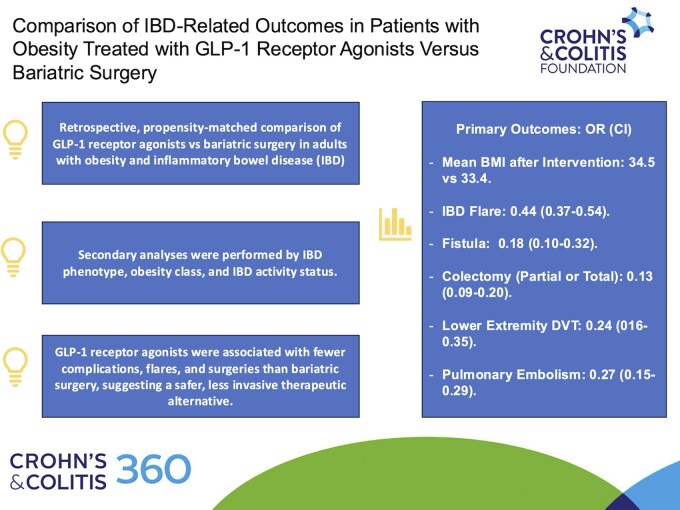

## Introduction

Obesity is now recognized as a significant factor influencing the pathogenesis and clinical course of IBD (inflammatory bowel disease).^1^^,^^2^ Approximately (15%-40%) of patients with IBD suffer from obesity, and it is associated with more severe disease activity, increased hospitalization rates, and a higher likelihood of surgical intervention.^1^^,^^2^

Obesity has been independently associated with increased disease activity and a higher risk of clinical relapse in both Crohn’s disease (CD) and ulcerative colitis (UC). In the IBD Partners cohort, which included more than 7000 patients, class II or III obesity was associated with greater odds of persistent disease activity or relapse in CD (aOR 1.86) and UC (aOR 2.97) when compared with patients with a normal BMI (body mass index).^3^

In addition to its association with disease activity, obesity has been shown to adversely affect multiple disease-specific outcomes. Patients with obesity and UC demonstrated a 72% increased risk of IBD-related hospitalization compared with patients with normal body weight, with each 1-kg/m^2^ increase in BMI associated with a 5% incremental increase in hospitalization risk.^4^

Mechanistically, adipose tissue is no longer viewed as a passive energy storage compartment but rather as an active endocrine and immune organ. In IBD, expansion of visceral and mesenteric adipose tissue, particularly the phenomenon of “creeping fat” in CD, has been implicated in the amplification of intestinal inflammation.^1^

Adipocytes and infiltrating immune cells within adipose tissue secrete a range of adipokines and cytokines, including tumor necrosis factor-α, interleukin-6, leptin, and resistin, which promote systemic and local pro-inflammatory signaling.^1^^,^^2^ Conversely, levels of adiponectin, an adipokine with anti-inflammatory properties, are often reduced in patients with obesity, further contributing to immune dysregulation.^2^ These alterations may enhance intestinal permeability, impair mucosal healing, and perpetuate chronic inflammation, thereby worsening IBD outcomes.

Once considered protective, excess adiposity is now understood to exert complex immunometabolic effects that may exacerbate intestinal inflammation and negatively influence disease outcomes.^1^

GLP1 RA (glucagon-like peptide-1 receptor agonist) drugs appear to be safe in patients with IBD and may provide both metabolic and disease-modifying benefits, with emerging evidence suggesting anti-inflammatory effects that extend beyond weight loss. Recent systematic reviews and meta-analyses have demonstrated that GLP-1 RAs are generally well tolerated in IBD populations, with gastrointestinal adverse events being the most commonly reported and typically manageable.^5^

Clinical data further support favorable disease-related outcomes among patients with IBD treated with GLP-1 RAs. A 2025 systematic review encompassing 14 studies reported no association between GLP-1 RA use and increased IBD exacerbations, while analyses from large registry-based cohorts demonstrated reduced risks of corticosteroid exposure, hospitalization, and surgical intervention.^5^

Obesity management encompasses a range of interventions, including pharmacotherapy, laparoscopic, and surgical options. BS (bariatric surgery) and GLP-1 RAs are two prominent strategies.^6–8^ However, GLP-1 RAs have never been compared to BS in patients with obesity and IBD.

This association prompted a growing interest in BS as a therapeutic strategy in this population.^1^^,^^2^ While several studies suggest that BS is generally feasible in selected patients with obesity and IBD and may confer metabolic and disease-related benefits, such as lower odds of IBD-related complications including fistula formation, suggesting a potential protective effect of sustained weight reduction on disease course,^9^ other studies suggest that BS is associated with procedure-specific risks, particularly in patients with IBD with active disease or immunosuppression. Alterations in gut anatomy, nutrient absorption, and microbiota, especially following Roux-en-Y gastric bypass, may predispose patients with IBD to infectious and postoperative complications, including Clostridioides difficile infection (C. diff).^10^^,^^11^

On the other hand, and beyond weight reduction, accumulating evidence suggests that GLP-1 RAs may exert direct anti-inflammatory and gut-protective effects relevant to IBD pathophysiology, mainly through modulating the immune responses, reducing pro-inflammatory cytokine production, enhancing epithelial barrier integrity, and influencing gut microbiota composition and intestinal motility.^12–15^ We explored the outcomes of using GLP-1 RA medications in the weight management of patients with obesity and IBD in comparison to undergoing BS for weight management in the same patient population.

## Methods

Clinical data were obtained from the TriNetX research network (Cambridge, MA, USA), a globally federated health research platform aggregating de-identified electronic medical record (EMR) data from over 200 healthcare organizations (HCOs) and more than 60 million patients in the United States. Participating HCOs include a mix of inpatient, outpatient, and specialty care settings, with most representing large academic health systems comprising main hospitals, satellite facilities, and outpatient clinics. All contributing HCOs remain anonymized.

Data were collected from 2006 through December 27, 2025, in accordance with TriNetX guidelines for retrospective cohort studies. Adult patients aged ≥18 years with IBD were identified using ICD-10 codes K50 (Crohn’s disease) or K51 (ulcerative colitis). Obesity was defined using ICD-10 code E66.9. BS was identified using relevant CPT, ICD-10 procedure codes, and clinical terminology codes, including sleeve gastrectomy, Roux-en-Y gastric bypass, and other bariatric procedures. Percentages for bariatric surgery procedures reflect the proportion of patients with specific procedure codes recorded in the database. All patients in the bariatric surgery cohort had a qualifying bariatric surgery procedure used to define cohort membership; however, individual procedure codes may appear at lower frequencies due to variations in coding practices and the use of multiple procedural classification systems.

For the primary analysis, patients with obesity and IBD were divided into two mutually exclusive cohorts: (1) patients who received GLP-1 RAs; dulaglutide, semaglutide, liraglutide, lixisenatide, exenatide, albiglutide, or tirzepatide and had no history of BS, and (2) patients who underwent BS but had no history of GLP-1 RA prescription.

To improve ascertainment of active medication use, patients in the GLP-1 RA cohort were required to have at least two prescriptions for any GLP-1 RA agent, at least 1 month apart.

The index date was defined as the date of the first qualifying GLP-1 RA prescription for the GLP-1 RA cohort and the date of BS for the surgical cohort.

Outcomes were defined using ICD-10 and prescription codes. Lower extremity deep vein thrombosis (LE DVT) was identified using ICD-10 code I82.4 (acute embolism and thrombosis of deep veins of the lower extremity), and pulmonary embolism (PE) using I26. C. diff colitis was defined by ICD-10 code A04.7 (enterocolitis due to *Clostridium difficile*), intestinal obstruction by K56.6 (other and unspecified intestinal obstruction), and constipation by K59.0. Colectomy was identified using ICD-10 procedure codes 0DBN (sigmoid colon excision), 0DBB (ileum excision), 0DBP (rectum excision), 0D8K (ascending colon excision), 0D8M (descending colon excision), 0D8H (cecum excision), 0D8E (large intestine excision), 0D8F (right large intestine excision), 0D8G (left large intestine excision), and 0D8A (jejunum excision). Abdominal pain was captured using R10.9, while fistula was defined as intestinal, rectal, or anal fistula using K63.2, K60.3, or K60.4, respectively. Nausea was defined by R11.0. IBD flare was defined as a new prescription of prednisone (Rx 8640), methylprednisolone (Rx 6982), infliximab (Rx 191831), cyclosporine (Rx 3008), upadacitinib (Rx 2196092), or a serum C-reactive protein (CRP) level ≥5 mg/L. Advanced therapies included prescriptions for ustekinumab, infliximab, azathioprine, secukinumab, upadacitinib, etanercept, tocilizumab, or golimumab, while steroid use was defined as a prescription of prednisone or methylprednisolone.

Secondary analyses included comparisons between GLP-1 RA and BS cohorts stratified by disease subtype (CD and UC), obesity class (class I: BMI 30-34.9; class II: BMI 35-39.9; class III: BMI ≥40), and disease activity. Active IBD was defined as a diagnosis of CD or UC in combination with a serum CRP level ≥5.0 mg/L.

Baseline characteristics, including demographics, comorbidities, IBD-related medications, prior surgical history, and common immune deficiency disorders, were assessed before and after propensity score matching (PSM). Patients with prior outcomes were excluded to ensure assessment of incident outcomes. This was done to ensure that only new (incident) outcomes occurring after exposure were analyzed, as including patients who had experienced these events before exposure would have compromised the causal interpretation of the results.

To account for confounding, 1:1 PSM was performed using a greedy nearest-neighbor algorithm based on 36 covariates, including age, sex, race, ethnicity, metabolic and cardiovascular comorbidities, immune deficiency disorders, and IBD advanced therapy medication prescription. Balance between cohorts was assessed using standardized mean differences.

Patients were followed from 1 day after the index date until the occurrence of the outcome of interest, last recorded encounter, or end of data availability. Mean follow-up duration was reported for each matched cohort.

Continuous variables were compared using independent *t*-tests, and categorical variables were compared using chi-square tests. Effect estimates were reported as odds ratio (ORs) with 95% confidence intervals (CIs). All statistical tests were two-sided with a significance threshold of α = 0.05. Analyses were conducted using the TriNetX Analytics platform.

## Results

A total of 12965 patients with obesity and IBD were identified in the GLP-1 RA cohort and 4177 patients in the BS cohort. The primary comparison assessed outcomes in patients with obesity and IBD who received GLP-1 RA therapy versus those who underwent BS.

**Figure 1:**
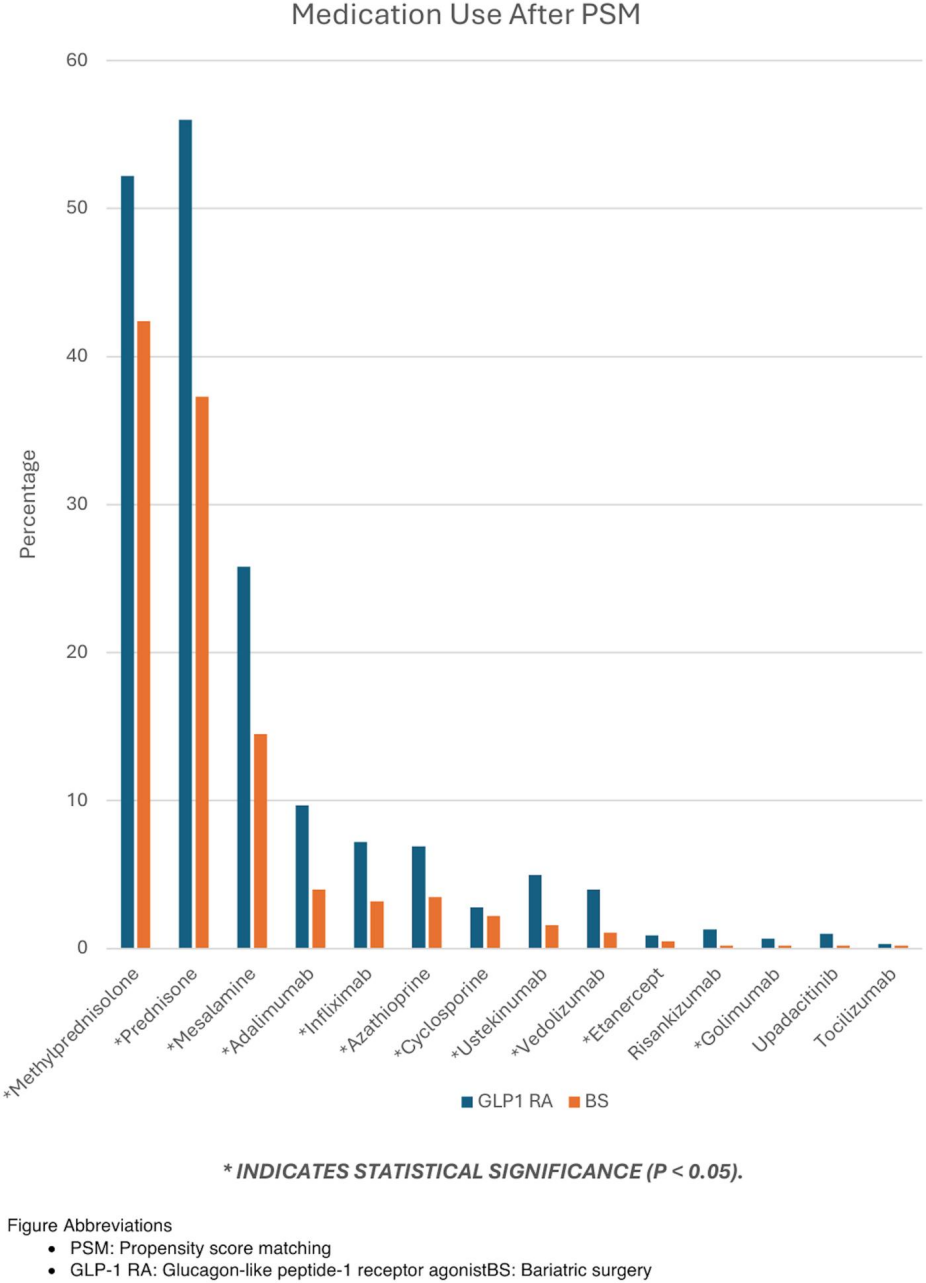
Rates of IBD–related medication use after propensity score matching. Comparison of post-matching medication utilization between patients with obesity and IBD treated with GLP1 RAs and those undergoing BS. PSM, propensity score matching; GLP-1 RA, glucagon-like peptide-1 receptor agonist; BS, bariatric surgery.

At baseline, patients receiving GLP-1 RA therapy were older (mean age 56.7 vs 52.9 years), with higher BMI (34.5 vs 33.4) compared to a baseline BMI of (37.1 ± 6.96 vs 36.2 ± 8.42), and had higher prevalence of type 2 diabetes mellitus (64.3% vs 31.5%), chronic kidney disease (19.6% vs 14.1%), and nicotine dependence (20.4% vs 19.1%), but lower rates of alcohol use disorder (2.3% vs 4.6%) and end-stage renal disease (2.0% vs 2.9%) compared with the BS cohort. Baseline IBD-related medication use, including corticosteroids (methylprednisolone 52.2% vs 42.4%, prednisone 56.0% vs 37.3%), immunomodulators (azathioprine 6.9% vs 3.5%, cyclosporine 2.8% vs 2.2%), and biologics (adalimumab 9.7% vs 4.0%, infliximab 7.2% vs 3.2%, ustekinumab 5.0% vs 1.6%, vedolizumab 4.0% vs 1.1%) was generally higher in the GLP-1 RA group, while prior BS history was limited to the BS cohort (Table 1, Figure 1).

**Table 1:** Baseline comparison (demographics, comorbidities, and medications) (IBD GLP1 RA vs IBD BS).

	Before matching			After matching	
	IBD with obesity on GLP-1 RA	IBD with obesity sp BS	*P* value	IBD with obesity on GLP-1 RA	IBD with obesity sp BS
**Total number of subjects**	12,965	4,177		3,478	3,478
**Age at index (yr)**	56.7 ±12.9	52.9 ± 13.3		53.5 ± 13.1	53.6 ± 13.2
**Female gender**	65.8%	81.4%	<.0001	81.9%	80.0%
**Race**					
**White**	72%	76.4%	.0054	78.6%	77.2%
**Black**	13.5%	15.2%	.0127	14.5%	14.9%
**Hispanic**	5.7%	15.2%	.041	6.21%	6.4%
**Asian**	1.3%	0.5%	<.0001	0.4%	0.5%
**Other**	2.5%	2.8%	.0176	2.5%	2.8%
**Comorbidities**					
** Diabetes mellitus**	64.3%	31.5%	<.0001	33.9%	33.2%
** Chronic kidney disease**	19.6%	14.0%	<.0001	13.3%	14.3%
** End stage renal**	2.0%	2.9%	.0008	2.3%	2.7
** Alcohol use disorder**	2.3%	4.6%	.1258	3.9%	3.3%
** Nicotine dependence**	20.4%	19.1%	.0341	18.7%	18.8%
** Anxiety**	45.3%	43.7%	.0240	45.5%	44.0%
** CVID**	0.31%	0.36%	.6944	0.2%	0.3%
** SCID**	0.07%	0.2%	—	0%	0.2%
**Medications**					
** Methylprednisolone**	52.2%	42.4%	<.0001	43.8%	43.7%
** Prednisone**	56.0%	37.3%	<.0001	39.7%	40.0%
** Mesalamine**	25.8%	14.5%	<.0001	16.3%	16.1%
** Adalimumab**	9.7%	4.0%	<.0001	4.6%	4.6%
** Infliximab**	7.2%	3.2%	<.0001	3.7%	3.6%
** Azathioprine**	6.9%	3.5%	<.0001	3.5%	3.8%
** Cyclosporine**	2.8%	2.2%	.0280	2.4%	2.3%
** Ustekinumab**	5.0%	1.6%	<.0001	1.9%	1.8%
** Vedolizumab**	4.0%	1.1%	<.0001	1.4%	1.3%
** Etanercept**	0.9%	0.5%	.0332	0.5%	0.6%
** Risankizumab**	1.3%	0.2%	—	0.4%	0.2%
** Golimumab**	0.7%	0.2%	.0010	0.3%	0.3%
** Upadacitinib**	1.0%	0.2%	—	0.2%	0.2%
** Tocilizumab**	0.3%	0.2%	—	0.2%	0.2%
**Surgical history**					
** Roux en Y (Laparoscopic)**	—	1.1%	<.0001	—	0.2%
** Roux en Y (Open)**	—	2.0%	—	—	0.2%
** Sleeve gastrectomy (laparoscopic)**	—	7.6%	<.0001	—	0.4%
** Sleeve gastrectomy (open)**	—	0.98%	—	—	0.2%
** Gastric banding (laparoscopic)**	—	0.5%	<.0001	—	0%
** Gastric banding (open)**	—	0.4%	<.0001	—	0%

Abbreviations: BMI, body mass index; BS, bariatric surgery; CKD, chronic kidney disease; CVID, common variable immunodeficiency; ESRD, end-stage renal disease; GLP-1 RA, glucagon-like peptide-1 receptor agonist; IBD, inflammatory bowel disease; SCID, severe combined immunodeficiency.

After propensity score matching, 3478 patients were included in each cohort. The mean follow-up duration was shorter in the GLP-1 RA group (648.8 vs 1346.2 days), and post-intervention BMI was slightly higher (34.5 vs 33.4 kg/m^2^, *P* < .0001) (Table 2).

**Table 2:** Outcomes after propensity score matching (IBD GLP1 RA vs IBD BS).

	IBD with obesity on GLP-1 RA	IBD with obesity sp BS	OR/CI	*P* **-value**
**Mean follow-up period (d)**	648.8	1346.2		
**Median follow-up period (d)**	471	1062		
**Mean BMI at baseline**	37.1 ± 6.96	36.2 ± 8.42		
**Mean BMI after intervention**	34.5	33.4		<.0001
**LE DVT (anytime)**	1.0%	4.2%	0.24 (0.16-0.35)	<.0001
**PE**	0.8%	2.9%	0.27 (0.17-0.41)	<.0001
**C. Diff Colitis**	0.7%	3.2%	0.22 (0.14-0.34)	<.0001
**Intestinal Obstruction**	1.2%	5.6%	0.21 (0.15-0.29)	<.0001
** Constipation**	8.1%	16.5%	0.44 (0.37-0.53)	<.0001
**Colectomy (Partial or Total)**	0.9%	6.5%	0.13 (0.09-0.20)	<.0001
**Abdominal Pain**	8.2%	20.6%	0.34 (0.28-0.42)	<.0001
**Fistula**	0.4%	2.2%	0.18 (0.10-0.32)	<.0001
**Nausea**	7.7%	16.5%	0.42 (0.35-0.50)	<.0001
**IBD Flare**	21.8%	38.3%	0.44 (0.37-0.54)	<.0001
**Need for Steroids**	18.8%	30.7%	0.52 (0.43-0.62)	<.0001
**Advanced Therapies**	1.9%	4.1%	0.45 (0.33-0.61)	<.0001

Abbreviations: BMI, body mass index; C. diff, Clostridioides difficile; CI, confidence interval; IBD, inflammatory bowel disease.; LE DVT, lower extremity deep vein thrombosis; OR, odds ratio; PE, pulmonary embolism.

Outcomes were defined as thromboembolic events (LE DVT, PE), gastrointestinal complications (C. diff colitis, intestinal obstruction, constipation, abdominal pain, nausea, fistula formation), surgical interventions (partial or total colectomy), and IBD-related disease activity (IBD flares, need for corticosteroids, initiation of advanced IBD therapies).

Compared with BS, GLP-1 RA therapy was associated with significantly lower rates of LE DVT (1.0% vs 4.2%; OR 0.24, 95% CI, 0.16-0.35) and PE (0.8% vs 2.9%; OR 0.27, 95% CI, 0.17-0.41). Gastrointestinal complications were also reduced: C. diff colitis 0.7% vs 3.2% (OR 0.22, 95% CI, 0.14-0.34), intestinal obstruction 1.2% vs 5.6% (OR 0.21, 95% CI, 0.15-0.29), constipation 8.1% vs 16.5% (OR 0.44, 95% CI, 0.37-0.53), abdominal pain 8.2% vs 20.6% (OR 0.34, 95% CI, 0.28-0.42), and nausea 7.7% vs 16.5% (OR 0.42, 95% CI, 0.35-0.50), all *P* < .0001. Surgical outcomes favored GLP-1 RA therapy, with lower rates of partial or total colectomy (0.9% vs 6.5%; OR 0.13, 95% CI, 0.09-0.20). Disease exacerbations were also reduced in the GLP-1 RA group: IBD flares 21.8% vs 38.3% (OR 0.44, 95% CI, 0.37-0.54), corticosteroid use 18.8% vs 30.7% (OR 0.52, 95% CI, 0.43-0.62), and initiation of advanced IBD therapies 1.9% vs 4.1% (OR 0.45, 95% CI, 0.33-0.61) (Table 2).

A sensitivity analysis was performed using a restricted outcome window from 1 day to 30 days after the index date to isolate early postoperative events and distinguish procedure-related risk of LE DVT and PE from longer-term effects. However, the number of observed events within this timeframe was insufficient to support meaningful statistical analysis.

CD: Among matched cohorts, the GLP-1 RA group had a mean BMI of (35.0 vs 33.7) compared to baseline BMI of (37.6 ± 7.21 vs 36.5 ± 8.18), and showed lower rates of thromboembolic events (DVT 0.9% vs 4.2%; PE 0.8% vs 3.2%), gastrointestinal complications (C. diff colitis 0.7% vs 3.5%; intestinal obstruction 1.9% vs 6.5%; constipation 8.2% vs 18.2%; abdominal pain 11.1% vs 22.1%; nausea 8.9% vs 19.2%), colectomy 1.1% vs 7.0%, and disease flares 21.6% vs 44.0%, despite higher post-intervention BMI (35.0 vs 33.7, *P* < .0001) and shorter follow-up (674.2 vs 1461.2 days) (Table 3).

**Table 3:** Outcomes after propensity score matching (CD GLP1 RA vs CD BS).

	CD with obesity on GLP-1 RA	CD with obesity sp BS	OR/CI	*P*-value
**Mean follow-up period (d)**	674.2	1461.2		
**Median follow-up period (d)**	502	1177		
**Mean BMI at baseline**	37.6 ± 7.21	36.5 ± 8.18		
**Mean BMI after intervention**	35.0	33.7		<.0001
**LE DVT**	0.9%	4.2%	0.21 (0.12-0.38)	<.0001
**PE**	0.8%	3.2%	0.26 (0.14-0.48)	<.0001
**C. Diff Colitis**	0.7%	3.5%	0.21 (0.11-0.39)	<.0001
**Intestinal Obstruction**	1.9%	6.5%	0.28 (0.18-0.43)	<.0001
** Constipation**	8.2%	18.2%	0.40 (0.31- 0.51)	<.0001
**Colectomy (partial or total)**	1.1%	7.0%	0.15 (0.09-0.26)	<.0001
**Abdominal pain**	11.1%	22.1%	0.44 (0.33-0.57)	<.0001
**Nausea**	8.9%	19.2%	0.41 (0.32-0.53)	<.0001
**IBD flare**	21.6%	44.0%	0.35 (0.26-0.47)	<.0001
**Need for steroids**	19.3%	34.9%	0.44 (0.34-0.57)	<.0001
**Advanced therapies**	2.82%	6.39%	0.42 (0.29-0.62)	<.0001

UC: Among matched cohorts, the GLP-1 RA therapy had a BMI of (34.6 vs 33.4) compared to a baseline of (37 ± 6.8 vs 35.9. ± 8.46), and showed lower thromboembolic events (DVT 1.1% vs 4.3%; PE 0.7% vs 2.5%), gastrointestinal complications (C. diff colitis 0.7% vs 3.3%; intestinal obstruction 0.8% vs 5.1%; constipation 7.0% vs 15.3%; abdominal pain 7.9% vs 19.1%; nausea 6.8% vs 14.8%), colectomy 0.9% vs 6.3%, and IBD flares 19.4% vs 35.4%, with higher BMI in the GLP-1 RA group (34.6 vs 33.4, *P* < .0001) and shorter follow-up (605.0 vs 1283.7 days) (Table 4).

**Table 4:** Outcomes after propensity score matching (UC GLP1 RA vs UC BS).

	IUC with obesity on GLP-1 RA	UC with obesity sp BS	OR/CI	*P*-value
**Mean follow-up period (d)**	605.0	1283.7		
**Median follow-up period (d)**	469	982		
**Mean BMI at baseline**	37 ± 6.8	35.9. ± 8.46		
**Mean BMI after intervention**	34.6	33.4		<.0001
**LE DVT**	1.13%	4.26%	0.25 (0.16-0.40)	<.0001
**PE**	0.7%	2.5%	0.27 (0.15-0.48)	<.0001
**C. Diff Colitis**	0.7%	3.3%	0.22 (0.13-0.39)	<.0001
**Intestinal obstruction**	0.78%	5.1%	0.14 (0.86-0.24)	<.0001
** Constipation**	7.0%	15.3%	0.41 (0.32-0.52)	<.0001
**Colectomy (partial or total)**	0.9%	6.3%	0.14 (0.09-0.24)	<.0001
**Abdominal pain**	7.9%	19.1%	0.36 (0.28-0.47)	<.0001
**Nausea**	6.8%	14.8%	0.42 (0.33-0.53)	<.0001
**IBD flare**	19.4%	35.4%	0.44 (0.34-0.56)	<.0001
**Need for steroids**	15.1%	29.4%	0.42 (0.33-0.54)	<.0001
**Advanced therapies**	1.1%	3.3%	0.31 (0.19-0.51)	<.0001

Obesity class I-III: Across matched cohorts, GLP-1 RA therapy consistently demonstrated lower gastrointestinal complications, colectomy rates, and IBD exacerbations. Post-intervention BMI differences varied by class but were generally higher or comparable in the GLP-1 RA group, while follow-up duration was shorter. Rare events, including thromboembolic complications and C. diff colitis, could not be reliably estimated in smaller subgroups due to low counts (Tables 5-7). Finally, among patients with active IBD, the GLP-1 RA therapy group had a BMI of (35.5 vs 33.7) compared to a baseline of (38.4 ± 7.47 vs 36.7 ± 8.76) was associated with lower gastrointestinal complications (intestinal obstruction 1.5% vs 7.2%; constipation 8.5% vs 21.9%; abdominal pain 10.7% vs 27.6%; nausea 7.5% vs 23.0%), fewer colectomies 1.7% vs 10.9%, and decreased IBD-related disease activity (steroid use 21.3% vs 44.5%; advanced therapy initiation 1.7% vs 7.5%), despite smaller BMI reduction (35.5 vs 33.7) and shorter follow-up (634.2 vs 1412.0 days) (Table 8).

**Table 5:** Outcomes after propensity score matching: IBD (obesity class I) GLP1RA vs BS (obesity class I).

	IBD with obesity class I on GLP-1 RA	IBD with obesity Class I sp BS	OR/CI	*P*-value
**Mean follow-up period (d)**	585.7	1342.0		
**Median follow-up period (d)**	444.5	1022.5		
**Mean BMI at baseline**	34.1 ± 4.45	35.2 ± 5.89		
**Mean BMI after intervention**	32.0	32.3		.143
**LE DVT**	Patient count was too small to run.			
**PE**	Patient count was too small to run.			
**C. Diff Colitis**	Patient count was too small to run.			
**Intestinal obstruction**	1.6%	4.7%	0.33 (0.17-0.65)	.0008
** Constipation**	6.9%	12.0%	0.54 (0.36-0.82)	.0034
**Colectomy (partial or total)**	1.89	5.4%	0.337 (0.17-0.64)	.0005
**Abdominal pain**	9.1%	17.8%	0.46 (0.30-0.69)	.0002
**Nausea**	7.4%	15.0%	0.45 (0.31-0.67)	<.0001
**IBD flare**	1.5%	3.9%	0.38 (0.19-0.77)	.0059
**Need for steroids**	20.9%	26.6%	0.73 (0.51-1.03)	.0736
**Advanced therapies**	1.1%	3.3%	0.31 (0.19-0.51)	<.0001

**Table 6:** Outcomes after propensity score matching: IBD (obesity class II) GLP1RA vs BS (obesity class II).

	IBD with obesity (Class II) on GLP-1 RA	IBD with obesity (Class II) sp BS	OR/CI	*P*-value
**Mean follow-up period (d)**	642.4	1345.8		
**Median follow-up period (d)**	477	1078.5		
**Mean follow-up (d)**	642.4	1345.8		
**Mean BMI at baseline**	37.5 ±4.81	38.7 ± 6.17		
**Mean BMI after intervention**	35.2	37.2		.143
**LE DVT**	Patient count was too small to run.			
**PE**	Patient count was too small to run.			
**C. Diff Colitis**	Patient count was too small to run.			
**Intestinal obstruction**	Patient count was too small to run.			
** Constipation**	6.7%	15.0%	0.40 (0.26-0.63)	<.0001
**Colectomy (partial or total)**	Patient count was too small to run.			
**Abdominal pain**	8.2%	18.9%	0.38 (0.24-0.60)	<.0001
**Nausea**	7.3%	17.0	0.38 (0.25-0.59)	<.0001
**IBD flare**	25.9%	42.3%	0.47 (0.31-0.72)	.0006
**Need for steroids**	21.9%	38.6%	0.44 (0.30-0.65)	<.0001
**Advanced therapies**	1.8%	3.8%	0.46 (0.2-1.002)	.045

**Table 7:** Outcomes after propensity score matching: IBD (obesity class III) GLP1RA vs BS (obesity class III).

	IBD with obesity (Class III) on GLP-1 RA	IBD with obesity (Class III) sp BS	OR/CI	*P*-value
**Mean follow-up (d)**	669.2	1479.9		
**Median follow-up period (d)**	517	1204		
**Mean BMI at baseline**	44.4 ± 7.24	45.1 ± 7.57		
**Mean BMI after intervention**	41.3	45.6		<.0001
**LE DVT**	Patient count was too small to run.			
**PE**	Patient count was too small to run.			
**C. Diff Colitis**	Patient count was too small to run.			
**Intestinal Obstruction**	Patient count was too small to run.			
** Constipation**	6.8%	14.3%	0.44 (0.27-0.71)	.0006
**Colectomy (partial or total)**	Patient count was too small to run.			
**Abdominal pain**	8.4%	21.2%	0.34 (0.20-0.55)	<.0001
**Nausea**	6.7%	14.0%	0.44 (0.27-0.71)	.0007
**IBD flare**	26.4%	42.5%	0.48 (0.3-0.78)	.0030
**Need for steroids**	22.5%	30.7%	0.65 (0.42-1.00)	.0513
**Advanced therapies**	2.0%	5.3%	0.37 (0.18-0.78)	.006

**Table 8:** Outcomes after propensity score matching: active IBD GLP1RA vs active IBD BS.

	Active IBD with obesity on GLP-1 RA	Active IBD with obesity sp BS	OR/CI	*P*-value
**Mean follow-up period**	634.2	1412.0		
**Median follow-up period (d)**	491	1153		
**Mean BMI at baseline**	38.4 ± 7.47	36.7 ± 8.76		
**Mean BMI after intervention**	35.5	33.7		<.0001
**LE DVT (anytime)**	0.9%	7.3%	0.11 (0.06-0.23)	<.0001
**PE**	Patient count was too small to run.			
**C. Diff Colitis**	0.9%	4.8%	0.18 (0.09-0.36)	<.0001
**Intestinal obstruction**	1.5%	7.2%	0.20 (0.12-0.35)	<.0001
** Constipation**	8.5%	21.9%	0.33 (0.24-0.45)	<.0001
**Colectomy (partial or total)**	1.7%	10.9%	0.14 (0.08-0.23)	<.0001
**Abdominal pain**	10.7%	27.6%	0.31 (0.22-0.44)	<.0001
**Fistula**	Patient count was too small to run			
**Nausea**	7.5%	23.0%	0.27 (0.19-0.37)	<.0001
**IBD flare**	Patient count was too small to run			
**Need for steroids**	21.3%	44.5%	0.33 (0.24-0.47)	<.0001
**Advanced therapies**	1.7%	7.5%	0.22 (0.13-0.37)	<.0001

Overall, GLP-1 RA were associated with improved gastrointestinal and disease-related outcomes compared with BS across IBD subtypes, obesity classes, and disease activity status, even in the context of smaller BMI reduction.

## Discussion

Obesity is increasingly recognized as a significant factor influencing the clinical course of IBD, contributing to more severe disease activity, increased hospitalizations, and a higher likelihood of surgical intervention.^1^^,^^2^ GLP-1 RA are a class of medications that mimic the action of the endogenous GLP-1 hormone, commonly used in the management of type 2 diabetes mellitus and obesity.^8^ Emerging evidence suggests that GLP-1 RAs may exert beneficial effects on patients with IBD independent of weight loss. Preclinical and clinical studies indicate that GLP-1 RA signaling modulates intestinal immune responses by directly acting on GLP-1 receptors expressed on gut intraepithelial lymphocytes, which in turn can reduce T cell-mediated inflammation and cytokine production, thereby ameliorating mucosal injury.^14^ GLP-1 RAs have also been shown to enhance epithelial barrier integrity and reduce oxidative stress in experimental colitis models, limiting immune activation and tissue damage.^13^ Furthermore, GLP-1 RA signaling appears to influence gut microbiota composition and gastrointestinal motility, both of which are implicated in IBD pathophysiology, suggesting a potential microbiome-mediated mechanism for reduced intestinal inflammation.^15^ Collectively, these mechanistic insights support the hypothesis that GLP-1 RAs may confer direct anti-inflammatory and mucosal-protective effects in patients with IBD, independent of their weight-lowering actions, providing a biologically plausible rationale for the improved disease outcomes observed in clinical studies.^12^

**Figure 2:**
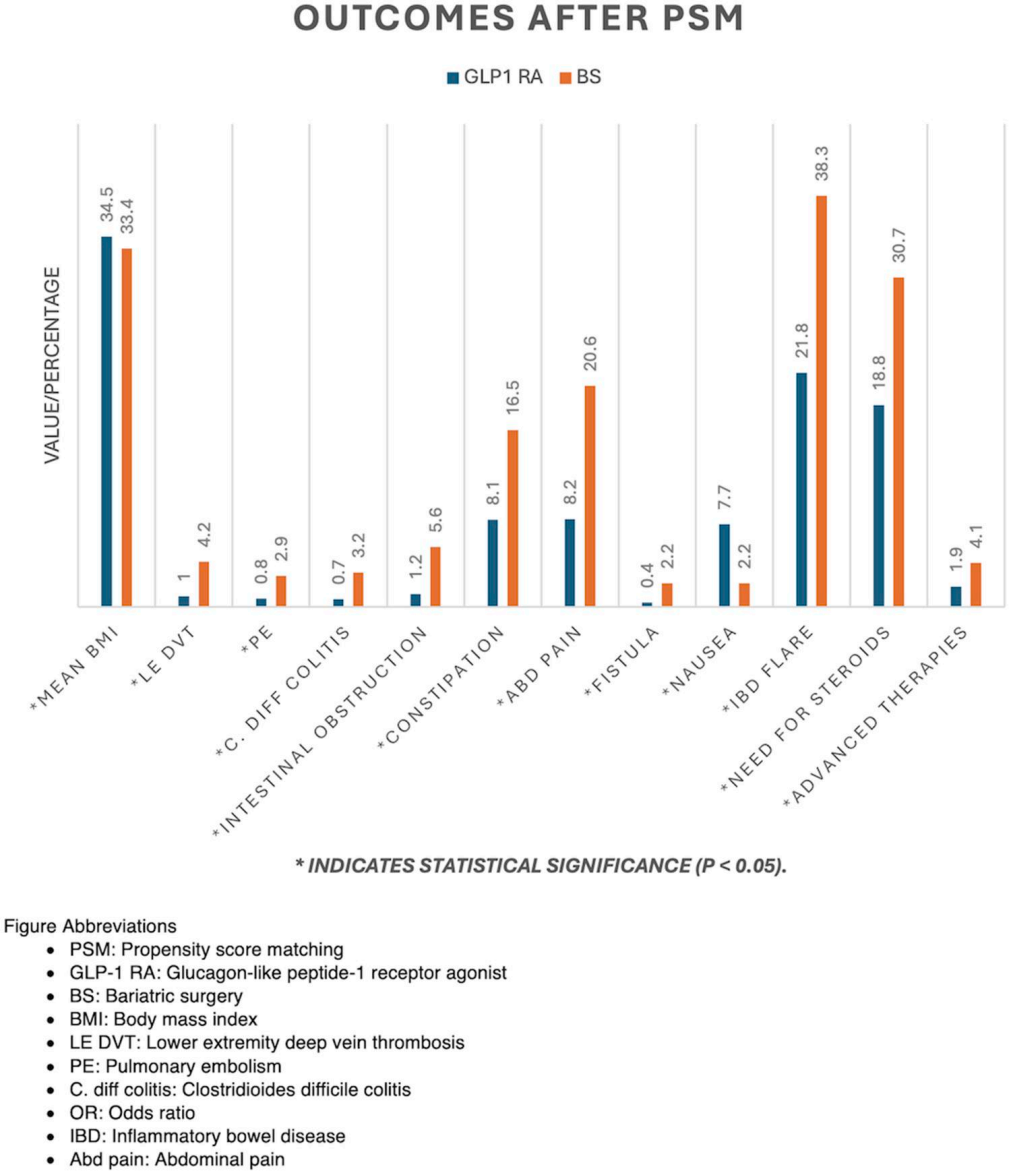
Clinical outcomes after propensity score matching. Comparison of clinical outcomes in patients with obesity and IBD treated with GLP1 RAs versus BS after propensity score matching. Effect estimates are presented as odds ratios. PSM, propensity score matching; GLP-1 RA, glucagon-like peptide-1 receptor agonist; BS, bariatric surgery; BMI, body mass index; LE DVT, lower extremity deep vein thrombosis; PE, pulmonary embolism; C. diff colitis, Clostridioides difficile colitis; OR, odds ratio; IBD, inflammatory bowel disease; Abd pain, abdominal pain.

These pleiotropic effects provide a biologically plausible explanation for the improved clinical outcomes observed in our study, including lower rates of hospitalizations, emergency department visits, and post-operative complications, independent of weight loss (Table 2, Figure 2).

When comparing patients with CD or UC and obesity treated with GLP-1 RA to those undergoing BS, we observed a significantly lower risk of IBD flare, defined by a serum CRP level ≥5 mg/L and initiation of prednisone or methylprednisolone, with an OR of 0.44 (Table 2). These findings remained consistent after subclassification by IBD subtype, with both CD and UC (Tables 3 and 4, respectively), and obesity classes I, II, and III (Tables 5-7) each showing a similar lower OR of IBD flare compared with their BS counterparts. The patient count for the active group was insufficient for analysis (Table 8).

Supporting these findings, a meta-analysis of 10 observational studies including 10,362 patients reported a pooled flare incidence of 14% among GLP-1 RA users, with no significant increase in corticosteroid use (OR 0.93, 95% CI, 0.17-5.16), treatment escalation (OR 0.70, 95% CI, 0.06-7.62), or IBD-related surgery (OR 0.32) compared to non-users.^16^ Additionally, a matched case-control study of 25 patients with IBD who underwent BS (median follow-up, 7.69 years) demonstrated numerically lower rates of rescue corticosteroid use (24% vs 52%; OR 0.36, 95% CI, 0.08-1.23) and IBD-related surgery (12% vs 28%; OR 0.20, 95% CI, 0.004-1.79) compared with matched controls, although these differences did not reach statistical significance.^17^

In addition to efficacy outcomes, we evaluated the incidence of GLP-1 RA-related gastrointestinal adverse events and the need for surgical intervention in patients with obesity and IBD. Patients treated with GLP-1 RAs demonstrated significantly lower odds of experiencing abdominal pain, nausea, and constipation compared with those undergoing BS (Table 2).

These findings remained consistent after subclassification by IBD subtype, including CD and UC (Tables 3 and 4), obesity classes I-III (Tables 5-7), and the active IBD group (Table 8), each showing a lower OR of GLP-1 RA-related adverse events compared with their BS counterparts. Consistent with these findings, a study of 36 non-diabetic patients with IBD receiving GLP-1 RAs reported nausea as the most common adverse event (31%), followed by constipation (25%).^18^

Notably, limited data exist regarding the incidence and severity of gastrointestinal symptoms following BS, specifically in patients with IBD, highlighting the need for further investigation in this population.

Similarly, the likelihood of requiring partial or total colectomy was substantially lower in the GLP-1 RA cohort (Table 2). Our findings suggest that GLP-1 RA therapy may be associated with a more favorable short-term gastrointestinal safety profile and a reduced need for major surgical intervention relative to BS in this population.

These results were consistent after subclassification by IBD subtype, including CD and UC (Tables 3 and 4), obesity class I (Table 5), and the active IBD group (Table 8), each showing a lower OR for colectomy compared with BS counterparts. The patient count for obesity classes II and III was insufficient for analysis (Tables 6 and 7).

A meta-analysis of 11 studies with 16,242 patients with IBD found that GLP-1 RA use was associated with lower risk of surgery, with a pooled log hazard ratio of 0.61 (95% CI, 0.44-0.84) and OR of 0.46 (95% CI, 0.32-0.67).^19^

On the other hand, BS shows mixed results regarding subsequent IBD-related surgical outcomes. One study reported that 15% of patients with IBD required surgical intervention for IBD following BS during a median 91-month follow-up.^17^

Our study found lower odds of requiring advanced therapies in patients with obesity and IBD treated with GLP-1 RAs compared with those undergoing BS (Table 2).

These findings were consistent after subclassification by IBD subtype, including CD and UC (Tables 3 and 4), obesity class II (Table 6), and the active IBD group (Table 8), with each showing lower ORs for the need for advanced therapies and corticosteroids compared with their BS counterparts. No difference in corticosteroid requirement was observed in obesity classes I and III (Tables 5 and 7).

A propensity-matched cohort study conducted in the United States found that BS, particularly sleeve gastrectomy, was associated with a reduced risk of IBD-related complications, including the need for intravenous steroids or subsequent IBD-related surgery, compared to non-surgical controls.^20^ Although this study was similar to ours in using the same database with a comparable patient sample and geographic distribution, it compared different types of BS and found favorable outcomes between sleeve gastrectomy and Roux-en-Y gastric bypass.

Our study further investigated fistula formation and intestinal obstruction rates between patients undergoing BS and those treated with GLP-1 RAs, variables that have not been previously explored (Table 2).

These results remained consistent after subclassification by IBD subtype, including CD and UC (Tables 3 and 4), obesity class II (Table 6), and the active IBD group (Table 8), each showing lower ORs for fistulae and intestinal obstruction compared with BS counterparts. No differences in fistula formation were noted in CD and UC patients or in obesity classes I and III (Tables 5 and 7). No differences in intestinal obstruction were observed in obesity classes II and III (Tables 6 and 7).

Of note, our study observed a relatively high proportion of steroid use among patients with obesity and IBD undergoing BS. Corticosteroids are known to impair wound healing and increase susceptibility to post-operative complications, including infections and anastomotic leaks.^21^ This could partially explain the differences in post-operative outcomes between patients treated with GLP-1 RAs and those undergoing BS, highlighting the need to consider concurrent steroid therapy when evaluating surgical risk in this population.

Sharma et al. analyzed data from the US National Inpatient Sample and found that prior BS was associated with a decreased incidence of fistula formation in patients with morbid obesity and IBD.^9^ Similarly, Weissman et al. demonstrated that obesity was independently associated with higher all-cause readmission rates, highlighting the burden of obesity as an independent disease modifier in IBD.^22^

We also found lower odds of C. diff colitis, LE DVT, and PE in patients treated with GLP-1 RAs compared with BS (Table 2, Figure 2). No differences were observed in LE DVT rates within the first 30 days post-index between the two groups.

These findings remained consistent after subclassification by IBD subtype, including CD and UC (Tables 3 and 4) and the active IBD group (Table 8), each showing lower ORs for DVT and PE compared with BS counterparts. No differences were observed in obesity classes I-III (Tables 5-7), and no difference in PE OR was noted for the active IBD group. Previous studies have shown that BS, particularly Roux-en-Y gastric bypass, is associated with a higher risk of C. diff colitis compared to sleeve gastrectomy, likely due to anatomical and microbiome alterations rather than weight loss alone.^10^^,^^11^

A major strength of our study is the use of a large, nationwide cohort derived from multiple healthcare organizations across the United States, providing robust statistical power and capturing geographic variability.

By leveraging the TriNetX database, we were able to analyze a diverse patient population, enhancing the generalizability of our findings. Furthermore, combining ICD-10 codes with IBD-specific medication prescriptions increases diagnostic accuracy, supporting the validity of our cohort.^23^

Additionally, our study provides novel insights into outcomes not previously explored, including LE DVT, PE, partial or total colectomy, fistulae, C. diff colitis, IBD flares, and use of advanced therapies, as well as comparative outcomes between GLP-1 RA and BS in patients with obesity and IBD (Figure 2).

We recognize several limitations, primarily due to the de-identified nature of the TriNetX database. This restricts the ability to perform manual chart reviews and limits our findings to data captured through ICD-10 codes. Reliance on ICD-10 coding may introduce misclassification bias, as diagnoses and comorbidities are only as accurate as the coding practices at participating sites. Furthermore, the database lacks detailed disease activity indices, limiting our ability to assess disease severity or fluctuations over time. Additionally, our definition of active IBD and flare incorporated CRP values; however, not all patients with active IBD mount a CRP response. As a result, disease activity may have been underestimated in some individuals, potentially leading to misclassification of active disease or flare status.

The mean follow-up duration was shorter in the GLP-1 RA cohort compared with the bariatric surgery cohort. This difference in follow-up time may have resulted in underestimation of outcome events in the GLP-1 RA group, potentially influencing the magnitude of the observed associations.

Procedural information is also limited, preventing a comprehensive evaluation of surgical techniques, perioperative factors, or longitudinal procedural outcomes. Missing data is present in certain clinical and laboratory variables, which may further affect the accuracy and generalizability of our findings.

Because patients with prior occurrences of the outcomes of interest were excluded, the findings may not be generalizable to individuals with a history of severe or recurrent IBD-related complications, potentially underestimating event rates in higher-risk populations.

Another important variable we could not assess, due to the limitations of the database, is the time to surgery.

Due to the inherent constraints of the TriNetX platform, our study is limited to observing associations and cannot establish causal relationships between interventions and outcomes; as such, outcomes could not be definitively attributed to BS versus underlying IBD. Specifically, we were unable to definitively distinguish whether certain events represented IBD-related complications or postoperative sequelae following BS. Additionally, detailed data on radiographic, endoscopic, and clinical measures of disease activity, as well as disease duration, were not available, limiting our ability to comprehensively assess baseline disease severity and its impact on outcomes.

Finally, given the extensive analytical framework involving eight separate comparisons, subgroup analyses by specific BS type were not performed.

Future research should include stratification by surgery type to better understand their differential impact on IBD outcomes.

In conclusion, the GLP-1 RA class of medications represents a promising therapeutic option for individuals with IBD, offering a noninvasive alternative to surgical interventions. This approach may potentially reduce complications and offer an alternative to weight loss in patients with IBD suffering from obesity. Our study indicates a favorable therapeutic profile, including improved disease outcomes and fewer complications.

Although our findings suggest that weight loss may improve outcomes in patients with IBD, we hope our research will serve as a foundation for further research. Specifically, future studies should explore the degree of weight loss required to achieve optimal IBD outcomes and what subtypes of patients benefit from weight loss medications and which from BS.

## Data Availability

The data used in this study were obtained from the TriNetX research network. Due to data use agreements and patient privacy protections, these data are not publicly available. Access to the TriNetX platform may be granted to qualified researchers at participating institutions upon reasonable request.
